# *Taenia solium* extracellular vesicles decrease reactive oxygen species production in human neutrophils by inhibiting myeloperoxidase activity

**DOI:** 10.1042/BSR20250355

**Published:** 2026-03-30

**Authors:** César Díaz-Godínez, Diana G. Ríos-Valencia, Andrea Toledo, Arturo Calderón-Gallegos, Patricia de la Torre, Karel Estrada, Raúl J. Bobes, Julio C. Carrero, Agnès Fleury, Juan P. Laclette

**Affiliations:** 1 Institute of Biomedical Research, Universidad Nacional Autónoma de México, Circuito M. de la Cueva, s/n, Ciudad Universitaria, Coyoacán C.P. 04510, CDMX, México; 2 School of Medicine, Universidad Nacional Autónoma de México, Ciudad Universitaria, Coyoacán C.P. 04510, CDMX, México; 3 Institute of Biotechnology, Universidad Nacional Autónoma de México, Av. Universidad 2001, Col. Chamilpa, CP 62210, Cuernavaca Mor., México

**Keywords:** extracellular vesicles, neutrophils, racemose cysticercus, ROS, Taenia solium

## Abstract

*Taenia solium* cysticerci are the etiological agents of neurocysticercosis, a leading cause of acquired epilepsy worldwide and a significant public health problem in endemic regions. The chronicity of the infection reflects a complex host–parasite interaction in which the parasite establishes long-term persistence through immunomodulatory mechanisms. In recent years, extracellular vesicles (EVs) have emerged as important mediators for some of these interactions. EVs are membrane-bound structures released by virtually every organism studied, which carry a diverse set of molecules that mediate intercellular communication. We isolated *Taenia solium* extracellular vesicles (TsEVs) from racemose cysticerci recovered from a patient, subsequently cultured *in vitro*, in order to characterize them by transmission electron microscopy, nanoparticle tracking analysis, and mass spectrometry-based proteomics. Effect of TsEVs on the production of reactive oxygen species (ROS) and myeloperoxidase (MPO) activity was evaluated in human neutrophils using fluorometric and colorimetric assays under basal and chemically stimulated conditions. Obtained TsEVs were under 200 nm in diameter. Proteomic analysis of four independent TsEV samples allowed identification of a core set of 336 proteins. Gene Ontology analysis of these vesicles was consistent with exosomes, as a number of exosome-associated marker proteins such as tetraspanins were identified. Proteins linked to immunomodulation and redox metabolism were also identified, suggesting a potential role in interactions with host cells. Finally, the effect of TsEVs on the respiratory burst of human neutrophils was evaluated, revealing a reduction in ROS production, primarily through inhibition of MPO activity, adding up evidence for the role of these vesicles in parasite’s survival against the host immune response.

## Introduction

Extracellular vesicles (EVs) are membrane-bound particles released from cells that can be classified according to their size into small and large EVs (limit at 200 nm) or according to their biogenesis into exosomes and ectosomes (originating from the endocytic system or cytoplasmic membrane outward budding, respectively) [1,2]. The study of EVs has grown significantly over the past decade, related to the regulation of various important biological processes, including intercellular communication, cell replication, metabolism, and immune modulation, among others [3–5]. Once released by cells, EVs are taken up by target cells, where they trigger a biological response or even regulate gene expression. The interaction of EVs with target cells may occur through surface molecules acting as ligands and subsequently signaling into the cell by delivering their cargo via membrane fusion or by endocytosis/phagocytosis [2]. The wide range of effects attributed to EVs is linked to the diversity of cargoes they can carry, either within their lumen or on their membrane. This diversity includes soluble and membrane-associated proteins, lipids, small metabolites, and a wide array of nucleic acids such as dsDNA, ssDNA, mtDNA, mRNA, miRNA, lncRNA, tRNA, and others [6–8]. Some molecules have been proposed as potential EV markers to distinguish their biogenesis, for instance, tetraspanins CD9, CD63, CD81, and CD82 [1]. However, the wide diversity of organisms that release EVs has so far precluded the identification of universal biomarkers for any particular type of EV.

EVs are involved in interactions between cells of the same organism as well as enable communication between different organisms [9]. This is particularly important for infections by pathogenic microorganisms, where released EVs contribute to the colonization and establishment of the pathogen by interacting with and modulating the host immune response. EVs from parasites such as *Entamoeba histolytica*, *Trichomonas vaginalis*, and *Trypanosoma cruzi* exert anti-inflammatory effects by down-regulating the innate immune response and promoting the production of anti-inflammatory cytokines, which may facilitate the establishment of infection [10–12]. In contrast, EVs from *Giardia intestinalis* and *Blastocystis sp*. led to increased intestinal permeability and a pro-inflammatory response linked to disease pathology [13,14].

*Taenia solium* is a cestode parasite that infects humans and pigs to complete its life cycle. The larval stage, also known as cysticercus, typically establishes in muscles, inner organs, and the brain of the intermediate porcine and human host, the last being the most severe form of this disease, known as neurocysticercosis (NCC) [15]. *Taenia solium* NCC is considered a neglected disease related to poor hygienic practices and poverty still prevalent in Asia, Sub- Saharan Africa, and Latin America. NCC is characterized by several neurological manifestations such as headache, seizures, neurological deficits, cognitive problems, and a compromise of the patient’s life [16,17]. The location, size, number, and stage of the cysticerci determine the type of clinical manifestation, with being NCC considered one of the main causes of acquired epilepsy in the world [18].

Racemose NCC is the extra-parenchymal variant of NCC with the highest morbidity and mortality rate in humans. In this case, the cysticercus appears as clusters of multiple vesicles that typically lack scolex. This pathology can lead to intracranial hypertension mainly related to hydrocephalus caused by obstruction of the cerebrospinal fluid circulation channels, arachnoiditis, vasculitis, and other complications derived from parasite growth [19]. Interestingly, recent studies have shown that in these forms of disease the interval between infection and diagnosis is approximately 20 yr [20]. This prolonged, largely asymptomatic period is probably due to the parasite’s immunosuppressive activity, which modulates the inflammatory response that would otherwise produce symptoms [21]. This situation contrasts sharply with the severe inflammatory reaction that accompanies parasite degeneration, either spontaneous or induced by cysticidal therapy, which can trigger severe manifestations and even become life-threatening. The immunosuppressive effect of *T. solium* cysticerci products, particularly miRNAs (typical components of EV cargo), on murine macrophages has been previously studied, demonstrating a decrease in pro-inflammatory cytokines such as IL-12, IL-16, and TNF, as well as nitric oxide synthase [22]. However, neutrophils (PMNs) are present in the granuloma surrounding parenchymal *T.solium* cysts and have been reported as a common cell type in the cerebrospinal fluid of patients with chronic extraparenchymal NCC harboring viable parasites [23–26]. Noteworthy, a previous study demonstrated that extracts of cysticerci were toxic to PMNs via reactive oxygen species (ROS) production, although the mechanisms involved were not studied [27]. In the present study, we described for the first time the release of EVs from the racemose form of two *T. solium* cysticerci, isolated from an NCC patient, and explored their immunomodulatory effect on the respiratory burst of human PMNs. Additionally, a proteomic analysis was performed in order to identify proteins that could help explain the obtained results.

## Materials and methods

### Collection and maintenance of *T. solium* cysticerci

Two subarachnoid racemose *T. solium* cysticerci were surgically removed from a patient with NCC at the National Institute of Neurology and Neurosurgery, Mexico City. The neurosurgical intervention was indicated solely for clinical reasons and was not influenced by research considerations. The parasite was recovered as part of routine medical care. Although prior approval from an ethics committee was not obtained, the present study corresponds to a single-patient case report and involved no additional interventions or risks beyond standard care. Written informed consent was obtained from the patient for the analysis of the recovered parasites and for the publication of the findings.

The racemose cysticerci were immediately transferred to RPMI-1640 medium (Gibco-Fisher Scientific) and maintained in culture as previously described, with minor modifications [28]. In brief, parasites were washed with sterile phosphate buffer solution (PBS; pH 7.4) with 1% antibiotics (100 U/ml penicillin and 100 mg/ml streptomycin) for 5 min and subsequently placed in 25 cm^3^ culture flasks with 10 ml of RPMI-1640 medium supplemented with 10% fetal bovine serum (FBS), 2 mM l-glutamine, 1% antibiotics, 1% non-essential amino acids, and 1% pyruvate (all reagents were purchased from Gibco-Fisher Scientific). The parasites were maintained for eight months at 37°C in a 5% CO_2_ humidified atmosphere with medium renewals every 6 ± 3 days. Photographs of both parasites were taken using a Leica MZ6 stereoscopic microscope. The Leica IM500 image manager program was used to capture the photographs (Imagic Bildverarbeitung).

### Isolation of *Taenia solium* extracellular vesicles

Culture medium was removed, and parasites were washed three times with sterile PBS for 1 min per wash to discard any debris. Subsequently, the parasites were placed in 2 ml of sterile RPMI-1640 medium supplemented with antibiotics and exosome-depleted FBS (Gibco-Fisher Scientific) and incubated for 6 h at 37°C in a 5% CO_2_ humidified atmosphere. After incubation, the culture supernatant was collected and processed for EVs isolation using Total Exosome Isolation (from cell culture media) reagent (Invitrogen) according to the manufacturer’s instructions. The *Taenia solium* extracellular vesicles (TsEVs) pellet was re-suspended in filtered PBS (pH 7.4), and the protein concentration was determined in a Nanodrop 2000 equipment (Thermo Fisher). TsEVs were isolated from cysticerci maintained in culture for 6–10 months.

### Transmission electron microscopy

The TsEVs pellet purified under the conditions described above was fixed with Karnovsky’s solution (2% paraformaldehyde and 2.5% glutaraldehyde) overnight at 4°C, carefully washed, and posteriorly preserved in 0.1 M sodium cacodylate buffer (pH 7.2) for 2 h at 4°C. Subsequently, the pellet was treated with osmium tetroxide (2%) in the dark for 2 h and washed twice with distilled water. The sample was then dehydrated and included in EPON 812 resin following standard procedures. Fine sections (1–2 μm) were stained with uranyl acetate and examined by transmission electron microscopy (TEM) (Jeol JEM 100C).

### Nanoparticle tracking analysis

Size determination of TsEVs was performed as we previously described with some modifications [10]. A sample of TsEVs was diluted 1:40 in filtered PBS (pH 7.4) and then analyzed with Nanosight NS3000 (Malvern Panalytical) equipment in three independent measurements.

### Mass spectrometry

For mass spectrometry, TsEVs were processed as described previously [10]. First, each sample (equivalent to 100 μg of protein) was run on 12% polyacrylamide gels just long enough to allow the mixture of proteins to enter the stacking gel and concentrate as a coarse band. After staining with Bio-Safe Coomasie G-250 stain (Bio-Rad), the band was cut and subjected to in-gel trypsin digestion. Briefly, gel pieces were washed with 50 mM ammonium bicarbonate (Acros) in 50% acetonitrile (Thermo Fisher), reduced with dithiothreitol (Acros), and alkylated with iodoacetamide (Sigma–Aldrich), washed again, and incubated overnight at 37°C in 75 μl solution of 6 ng/μl trypsin (trypsin gold; Promega). The resulting peptides were extracted using solutions of 50% and 80% acetonitrile (ACN) with 0.5% formic acid (Millipore), and the recovered solution was dried in a vacuum concentrator.

Dried peptides were dissolved in 60 μl of 0.1% trifluoroacetic acid (TFA; Sigma–Aldrich), and desalted using 2-core MCX stage tips (3M, 2241) (Rappsilber et al., 2003). The stage tips were activated with ACN, followed by 3% ACN with 0.1% TFA. Next, samples were applied, followed by two washes with 3% ACN with 0.1% TFA and one wash with 65% ACN with 0.1% TFA. Peptides were eluted with 75 μl of 65% ACN with 5% NH_4_OH (Sigma) and dried.

### LC-MS methods

Samples were dissolved in 25 μl of water containing 2% ACN and 0.5% formic acid. Then, 2 μl (0.5 μg) was injected onto a pulled-tip nano-LC column with a 75 μm inner diameter packed to 25 cm with 3 μm, 120 Å, C18AQ particles (Dr. Maisch). The peptides were separated using a 120 min gradient from 3%–28% ACN, followed by a 7 min ramp to 85% ACN and a 3 min hold at 85% ACN. The column was connected in line with an Orbitrap Lumos via a nanoelectrospray source operating at 2.2 kV. The mass spectrometer was operated in data-dependent top speed mode with a cycle time of 2.5 s. MS1 scans were collected at 120,000 resolution with a maximum injection time of 50 ms. Dynamic exclusion was applied for 15 s. HCD fragmentation was used, followed by MS2 scans in the ion trap with a 35 ms maximum injection time.

### Database searching and label-free quantification

For graphical representation of relative protein abundance, values obtained by Label-Free Quantitation using Proteome Discoverer (v3.2) were transformed using log_2_ (*x* + 1) and subsequently standardized by *z*-score normalization to a symmetrical range from −3 to +3. Data processing and visualization were performed in Python (v3.12) using the pandas, seaborn, and matplotlib libraries. A hierarchical clustering heatmap was generated with a divergent color scale, where blue and red tones represent proteins of lower or higher relative abundance, respectively.

### Gene Ontology annotation and functional classification

Protein identifiers obtained from the proteomic analysis of TsEVs were manually curated to select the proteins consistently identified across the four biological replicates using the Venny (v2.1) tool. Corresponding protein sequences were retrieved from the *T. solium* genome database (NCBI accession: GCA_001870725.1) using custom Python scripts that extracted the FASTA entries based on accession numbers. Functional annotation was performed with Argot (v2.5), and only Gene Ontology (GO) terms with a total score ≥50 were considered for downstream analyses to reduce spurious assignments. Data integration and filtering were conducted with Python using the pandas library, and the top 20 enriched categories per ontology were used for visualization.

Hypothetical proteins ranking within the top 25 most abundant proteins (based on relative abundance) were selected for further analysis. To explore potential homology, each sequence was analyzed using the BLASTp algorithm against the non-redundant protein database at NCBI. The top 20 protein sequences with the highest percentage of identity were selected for subsequent phylogenetic analyses. Multiple sequence alignments were generated in MEGA (v12) software, and phylogenetic trees were constructed within the same platform using the maximum likelihood method. To further investigate functional features, the five sequences with the highest identity scores were subjected to domain and family analysis using the InterPro database. Conserved regions and putative protein signatures were identified and annotated. The resulting alignments were subsequently exported and refined using Jalview (v2.11.5.0) for visualization and figure preparation.

### Neutrophil isolation

PMNs were isolated from the peripheral blood of healthy volunteers using a Ficoll-Paque™ gradient (GE Healthcare) and a hypertonic shock to lyse erythrocytes [29]. The cells were re-suspended in PBS (pH 7.4), counted using a hemocytometer, and stored at 4°C until use.

### ROS detection in human PMNs

To quantify ROS, we followed our previously described methodology with some modifications [30]. In brief, PMNs (5 × 10^5^) were re-suspended in 500 μl of PBS supplemented with 10 μM 2′,7′-dichlorofluorescein diacetate (H_2_DCFDA; Merck) and incubated for 30 min at 37°C in the dark. The cells were then centrifuged at 4000 rpm for 2 min and resuspended in 500 μl of RPMI-1640 supplemented with 5% FBS. Subsequently, 100 μl of the suspension (1 × 10^5^ PMNs) was transferred to a 96-well plate and allowed to settle for 10 min at 37°C. TsEVs, corresponding to 1 μg of protein, were added to the H_2_DCFDA-treated PMNs, followed by immediate stimulation (or no stimulation) with phorbol 12-myristate 13-acetate (PMA, 50 nM, Sigma–Aldrich), or the calcium ionophore A23187 (10 μM, Sigma–Aldrich). Unstimulated PMNs were included as a control for basal ROS production. Fluorescence intensity was measured after 1 h of incubation at 37°C from the bottom of the well using a Synergy HTX spectrofluorometer (BioTek) with excitation and emission filters of 485 and 528 nm, respectively.

### Superoxide anion detection

PMNs (1 × 10^5^) were placed in 1.5 ml tubes in a final volume of 80 μl of RPMI-1640 medium. Nitroblue tetrazolium (NBT; 0.05% in PBS; Sigma–Aldrich) was added (40 μl per tube), followed by TsEVs at a concentration equivalent to 1 μg of protein, in the presence or absence of PMA (10 μM). Unstimulated PMNs were included as a control for basal superoxide production. The samples were incubated at 37°C for 2 h in the dark. After incubation, they were centrifuged at 8300×***g*** for 5 min, and the supernatant was discarded to remove unreacted NBT. The cell pellets were re-suspended in 120 μl of 2 M KOH, followed by the addition of 140 μl of dimethyl sulfoxide (Sigma–Aldrich) to solubilize the formazan crystals generated by NBT reduction. Finally, 100 μl of the solution was transferred to a 96-well plate, and the absorbance was measured at 620 nm using a plate reader Multiskan FC (ThermoScientific).

### Mitochondrial superoxide detection

Detection of mitochondrial superoxide anion in human PMNs was carried out as described previously with minor modifications [30]. In brief, PMNs (4 × 10^5^) were re-suspended in 400 μl of PBS supplemented with MitoSOX™ Red (10 μM; Invitrogen) and incubated for 30 min at 4°C in the dark. The cells were then centrifuged at 1300×***g*** for 2 min and re-suspended in 400 μl of RPMI-1640 medium. Next, 100 μl of MitoSOX-pretreated PMNs (1 × 10^5^) were transferred to a 96-well plate and allowed to settle for 20 min. TsEVs equivalent to 1 μg of protein were added, and the cells were either immediately stimulated or not with A23187 (10 μM). Fluorescence was measured from the bottom of the wells after 2 h using a Synergy HTX spectrofluorometer with excitation and emission filters of 485 and 580 nm, respectively.

### Intracellular hydrogen peroxide detection assay

A commercial kit for intracellular hydrogen peroxide assay (Sigma–Aldrich) was used following the manufacturer’s instructions with some modifications. Briefly, 4 × 10^5^ PMNs were re-suspended in 400 μl of fluorescent peroxide sensor and incubated for 30 min at 37°C. Posteriorly, PMNs were washed three times with PBS (pH 7.4) and re-suspended in 400 μl of RPMI-1640 medium supplemented with 5% FBS. Subsequently, 100 μl of the suspension (1 × 10^5^ PMNs) was transferred to a 96-well plate and allowed to settle for 20 min. TsEVs equivalent to 1 μg of protein were added, and immediately the cells were either stimulated or not with PMA (50 nM) or A23187 (10 μM). Fluorescence was measured from the bottom of the wells after 1 h using a Synergy HTX spectrofluorometer with excitation and emission filters of 485 and 580 nm, respectively.

### Myeloperoxidase activity detection assay

Myeloperoxidase (MPO) activity was determined as we described previously [30]. In brief, PMNs (5 × 10^5^) were re-suspended in 500 μl of RPMI-1640 medium supplemented with 5% FBS and luminol (200 μM; ChemCruz). Each 100 μl of cell suspension (1 × 10^5^ PMNs) was placed in a 96-well plate and incubated for 20 min at 37°C for sedimentation. TsEVs equivalent to 1 μg of protein were added, and immediately the cells were either stimulated or not with PMA (50 nM) or A23187 (10 μM). Luminescence was measured from the bottom of the wells (after 15 min for A23187 and 2.5 h for PMA) using a Synergy HTX spectrofluorometer.

### Statistical analysis

Graphs represent the mean ± standard deviation. Data were analyzed using GraphPad Prism 8.0.1 software, employing one-way ANOVA with Tukey’s post hoc test or the Kruskal–Wallis test followed by Dunn’s post hoc correction for multiple comparisons. A *P*-value ≤0.05 was considered statistically significant.

## Results

### Characterization of TsEVs size and morphology

**Figure 1:**
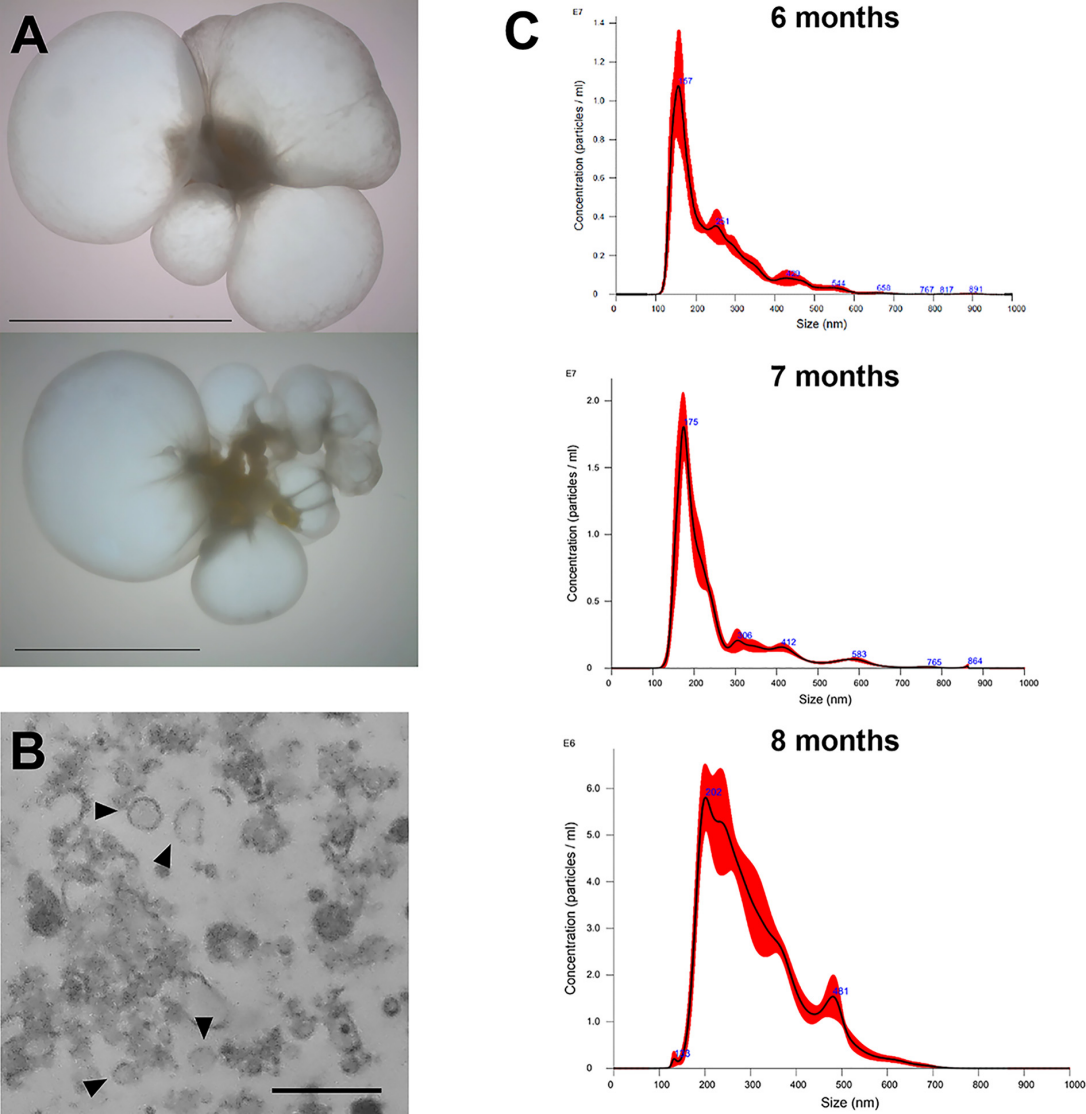
Characterization of EVs derived from *Taenia solium* (TsEVs) (**A**) Representative images of the two racemose cysticerci cultured *in vitro* for the present study. Scale bar represents 10 mm. (**B**) TsEVs were isolated from the culture supernatant, fixed, and contrasted for TEM visualization. Micrographs showed spherical, membrane-bound vesicles (black arrowheads). Scale bar represents 500 nm. (**C**) NTA of TsEVs collected after 6, 7, and 8 months of *in vitro* culture of cysts.

In order to determine the presence of TsEVs in the pellet purified from the two racemose cysticerci (Figure 1A), we carried out morphological observations by TEM. The analysis revealed membrane-delimited, round nanostructures compatible with EV morphology, predominantly within the 100–200 nm range (Figure 1B). Moreover, nanoparticle tracking analysis (NTA) was performed at three different time points of the two cysticerci cultures: 6, 7, and 8 months after the surgical isolation. As shown in Figure 1C, TsEVs of 6 months’ culture displayed a modal diameter of 157 nm, with larger vesicles of 251, 420, and 544 nm also detected. TsEVs obtained one month later were slightly larger, with a modal diameter of 175 nm. The third sample of TsEVs obtained the following month revealed a notable increase in size, showing a modal diameter of 202 nm and another population of vesicles 481 nm in diameter.

### TsEVs proteome

**Figure 2:**
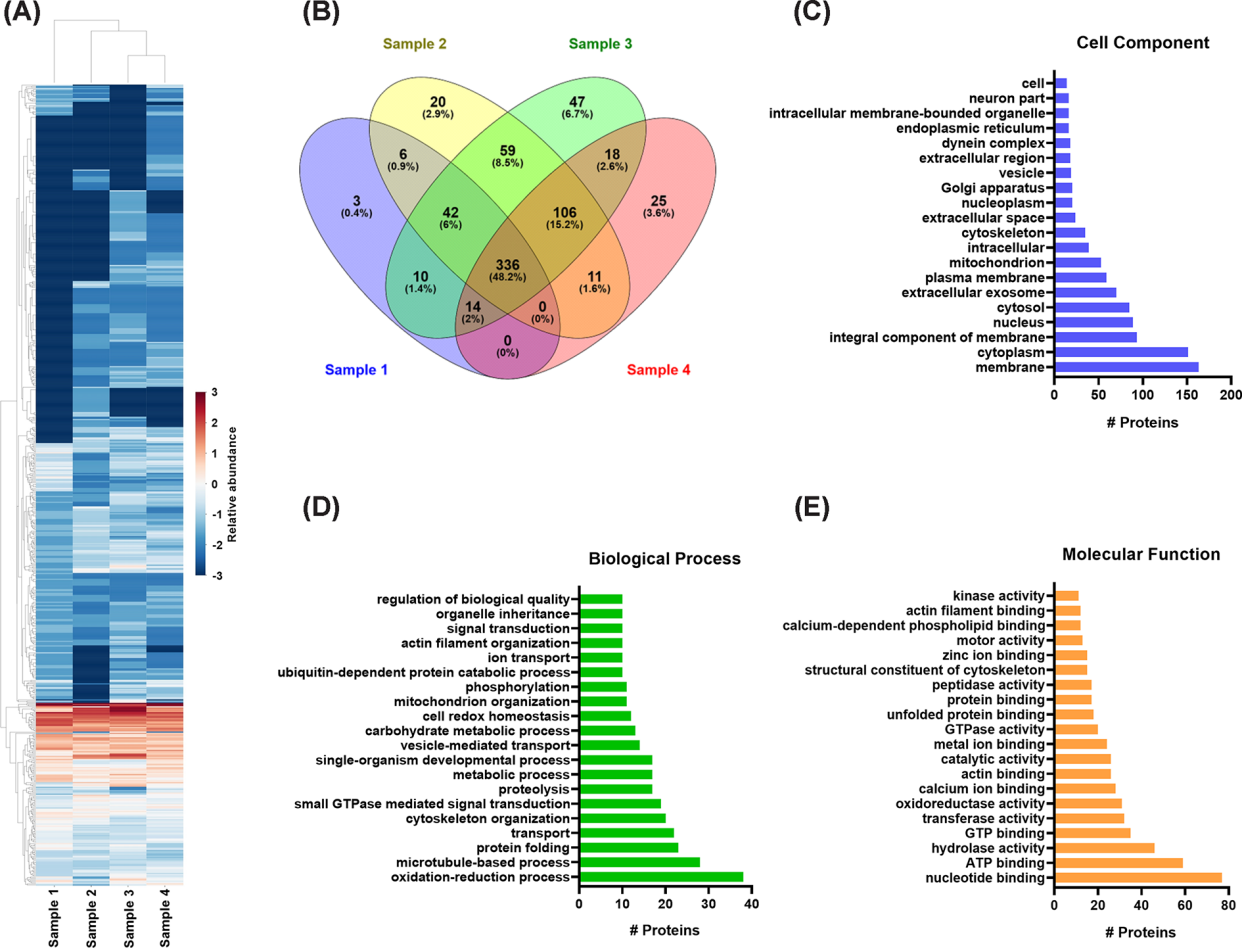
Proteomic analysis and functional annotation of TsEVs isolated from racemose cysticerci (**A**) Heatmap of the proteomic analysis on four independent TsEV preparations, showing differential abundance patterns among samples. The corresponding relative *z*-scores are displayed. Red tones indicate higher *z*-scores, reflecting greater relative abundance for each protein, whereas blue tones represent proteins with the lowest abundance. (**B**) Venn diagram representing the number of shared and unique proteins among the four TsEV preparations. GO analysis of proteins identified in TsEVs, classified according to (**C**) cellular component, (**D**) biological process, and (**E**) molecular function.

Proteomic analysis of four TsEV samples collected over time from two racemose cysticerci maintained in culture for eigth months was performed. The Cluster heatmap and dendrogram revealed that samples 3 and 4 exhibited the most similar abundance patterns, showing moderate resemblance to sample 2, whereas sample 1 displayed the greatest divergence (Figure 2A). The upper cluster of the heatmap encompasses a large group of proteins, representing approximately three-quarters of the total, showing low relative abundance (blue tones). Within this region, proteins absent in specific samples (dark blue tones) are also evident, indicating high heterogeneity among samples. The intermediate cluster, characterized by red tones, corresponds to a group of proteins consistently abundant across all four TsEVs samples, exhibiting comparable relative levels throughout the sampling period. In the lower portion of the heatmap, a cluster of proteins with medium/low abundance was observed. These proteins also appeared uniformly represented across all samples.

A total of 697 proteins were identified, of which 411 proteins were detected in sample 1, 580 in sample 2, 632 in sample 3, and 510 in sample 4. Comparative analysis using a Venn diagram (Figure 2B) showed that 336 proteins (48.2%) were shared among all four replicates, constituting a common core of the TsEV proteome. Moreover, a high proportion of proteins, between 350 and 543, were shared between pairs of replicates. These results demonstrate consistency in the protein repertoire across the four replicates. Finally, few exclusive proteins were observed in each sample: 3 (0.4%) were unique to sample 1, 20 (2.9%) in sample 2, 47 (6.7%) in sample 3, and 25 (3.6%) in sample 4.

GO analysis according to cellular components (Figure 2C) showed that the largest proportion of TsEVs proteins correspond to the parasite’s membrane and cytoplasm components. As expected, terms associated with extracellular exosomes were present, ranking sixth in representativeness, consistent with the origin of the samples. Proteins associated with the cytosol, nucleus, mitochondria, and endoplasmic reticulum were also detected, suggesting the involvement of multiple cellular compartments in the proteomic composition of TsEVs. Noteworthy, GO according to biological processes (Figure 2D) showed oxidation-reduction, microtubule-dependent processes, protein folding, and intracellular transport as the most representative categories, together with less represented small GTPase-mediated signaling and catabolic pathways, indicating that TsEVs’ cargo is related to cellular homeostasis and vesicular trafficking regulation. Regarding GO molecular functions (Figure 2E), categories related to nucleotide and ATP binding and hydrolase, transferase, and oxidoreductase activities were predominant.

Classical EV markers such as actin isoforms, tubulin, annexin, and heat shock cognate 70 kDa protein were present among the 25 most abundant TsEVs proteins (Table 1). Other recognized EV proteins identified include major vault protein, phosphoenolpyruvate carboxykinase GTP, cytoplasmic malate dehydrogenase, 14-3-3 protein zeta, calpain-A, and filamin-A. Interestingly, paramyosin, a protein that has been involved in complement cascade inhibition, was also among the top list. Moreover, three hypothetical proteins with accession codes KAL5962132.1, KAL5967266.1, and KAL5970357.1 ranked first, third, and eighth in abundance, respectively.

**Table 1:** Top 25 most abundant proteins in TsEVs (average of 4 replicates)

Ranking	Identified protein	Accession	Molecular weight	Average abundance^*^	Reported in EVs
1	Hypothetical protein	KAL5962132.1	67 kDa	126.75 ± 12.8	No (hypothetical)
2	Major vault protein	KAL5969712.1	97 kDa	100.00 ± 46.6	Yes (detected in some EV proteomes)
3	Hypothetical protein	KAL5967266.1	33 kDa	90.75 ± 27.1	No (hypothetical)
4	Phosphoenolpyruvate carboxykinase GTP	KAL5961700.1	70 kDa	75.75 ± 16.4	Yes (detected in some EV proteomes)
5	Actin cytoplasmic 2	KAL5965600.1	42 kDa	67.00 ± 8.8	Yes (classical EV marker)
6	Tubulin beta-2 chain	KAL5969386.1	50 kDa	59.75 ± 29.6	Yes (classical EV marker)
7	Actin	KAL5964705.1	42 kDa	56.50 ± 9.7	Yes (classical EV marker)
8	Hypothetical protein	KAL5970357.1	28 kDa	55.75 ± 3.8	No (hypothetical)
9	Tubulin beta chain	KAL5969061.1	50 kDa	55.75 ± 34.6	Yes (classical EV marker)
10	Tubulin beta chain	KAL5968764.1	50 kDa	52.75 ± 31	Yes (classical EV marker)
11	Heat shock cognate 70 kDa protein	KAL5962938.1	71 kDa	50.50 ± 11.8	Yes (classical EV marker)
12	Malate dehydrogenase cytoplasmic	KAL5963499.1	36 kDa	50.25 ± 14.2	Yes (detected in some EV proteomes)
13	Tubulin beta chain	KAL5962583.1	50 kDa	50.25 ± 31.1	Yes (classical EV marker)
14	Actin-1	KAL5963667.1	31 kDa	49.00 ± 11.2	Yes (classical EV marker)
15	14-3-3 protein zeta	KAL5969180.1	28 kDa	47.25 ± 10	Yes (detected in some EV proteomes)
16	Annexin A13	KAL5960689.1	35 kDa	46.00 ± 17	Yes (classical EV marker)
17	Calpain-A	KAL5965764.1	93 kDa	41.00 ± 14.7	Yes (detected in some EV proteomes)
18	Annexin A7	KAL5960686.1	41 kDa	41.00 ± 14.6	Yes (classical EV marker)
19	Filamin-A	KAL5967554.1	182 kDa	39.00 ± 9	Yes (detected in some EV proteomes)
20	Hydatid disease diagnostic antigen P-29	KAL5966573.1	27 kDa	38.50 ± 25.8	No (parasite antigen)
21	Alpha-actinin sarcomeric	KAL5966186.1	104 kDa	37.50 ± 7	Yes (classical EV marker)
22	Tubulin alpha-1B chain	KAL5963602.1	54 kDa	37.50 ± 12.4	Yes (classical EV marker)
23	Annexin A13	KAL5968958.1	36 kDa	36.75 ± 5.6	Yes (classical EV marker)
24	Paramyosin	KAL5966408.1	108 kDa	36.00 ± 30.4	Yes (detected in some EV proteomes)
25	Tubulin beta-1 chain	KAL5961394.1	50 kDa	34.25 ± 20.8	Yes (classical EV marker)

Data are presented as mean abundance values ± standard deviation.

* Relative abundance calculated by Label-Free Quantitation (LFQ).

**Figure 3:**
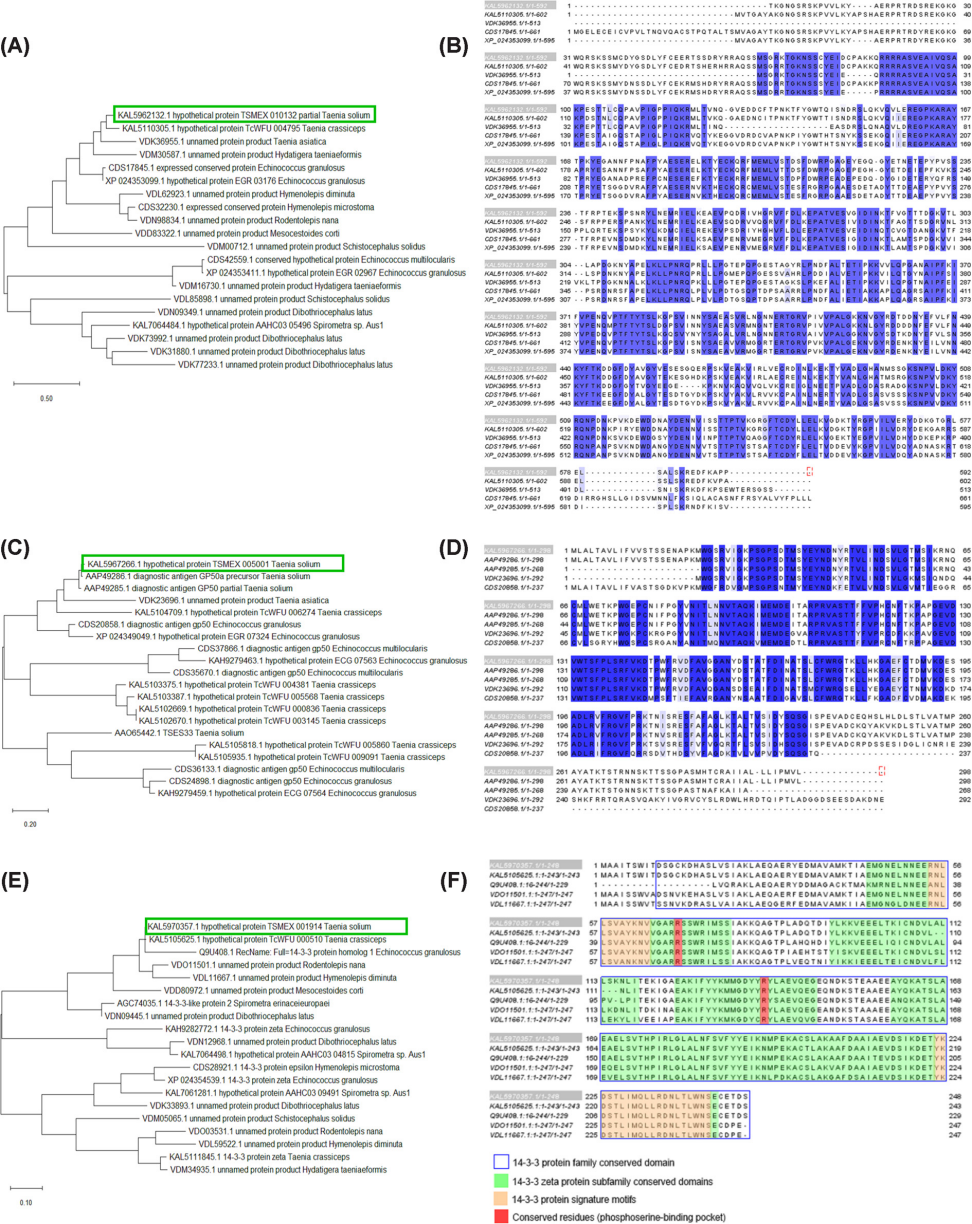
Phylogenetic analysis and sequences alignment of highly abundant hypothetical proteins in TsEVs (**A**) Phylogenetic analysis of the hypothetical protein KAL5962132.1. (**B**) Sequence alignment of KAL5962132.1 with the five most similar proteins; conserved regions are highlighted in purple. (**C**) Phylogenetic analysis of the hypothetical protein KAL5967266.1. (**D**) Sequence alignment of KAL5967266.1, showing conserved regions (purple). (**E**) Phylogenetic analysis of the protein KAL5970357.1, placed within the clade of cestode 14-3-3 proteins. (**F**) Sequence alignment of KAL5970357.1, showing conserved motifs and residues characteristic of the 14-3-3 protein family.

Since these hypothetical proteins were among the most abundant in all four samples, we performed a dedicated analysis to try to determine their identity and possible function. Phylogenetic analysis of the hypothetical protein KAL5962132.1 showed no close relationship with proteins of characterized function and appeared grouped with other hypothetical and unnamed proteins from cestodes (Figure 3A). Sequence alignment with the five highest identity proteins revealed highly conserved regions, suggesting the existence of common structural motifs despite the absence of functional annotation (Figure 3B). Similarly, the hypothetical protein KAL5967266.1 clustered within a clade with a *T. solium* diagnostic antigen GP50 and uncharacterized proteins of other cestodes (Figure 3C), showing highly conserved regions without functional domains or motifs in multiple sequence alignments (Figure 3D). In contrast, protein KAL5970357.1 was placed within the clade of 14-3-3 proteins of cestodes, closely associated with a characterized *Echinococcus granulosus* orthologue (Figure 3E). Multiple sequence alignment (Figure 3F) identified the characteristic domains of the 14-3-3 protein family, specifically those associated with the signaling proteins, 14-3-3 zeta subfamily, as well as the sequence motifs and conserved residues that form the phosphoserine-binding pocket, thereby confirming protein KAL5970357.1 as a functional member of this group of regulatory proteins.

### TsEVs display classical extracellular vesicle markers

Beyond the 25 most abundant proteins, several characteristic markers of EVs were identified and found consistently in all four biological replicates (Table 2). Among them were tetraspanins 6 and 7, both considered canonical exosome markers directly related to vesicle biogenesis. Also included were cytosolic proteins with lipid or membrane affinity, such as the abovementioned heat shock cognate 70 kDa protein, heat shock protein HSP90-alpha, and several annexin isoforms (A6, A7, A8, and A11); structural cytosolic proteins such as actins, filamin-A, tubulins, and glyceraldehyde-3-phosphate dehydrogenase.

**Table 2:** EV markers outside top 25, present in the four replicates

Group marker	Protein	Accession	Molecular weight
Cytosolic proteins in EVs with lipid or membrane protein-binding ability	Heat shock cognate 70 kDa protein	KAL5962473.1	42 kDa
	Heat shock protein HSP90-alpha	KAL5962474.1	79 kDa
	Annexin A6	KAL5970641.1	40kDa
	Annexin A7	KAL5960686.1	41 kDa
	Annexin A8	KAL5968959.1	38 kDa
	Annexin A8	KAL5967627.1	39 kDa
	Annexin A11	KAL5969575.1	47 kDa
Cytosolic proteins in EVs; promiscuous incorporation in EVs	Actin	KAL5967672.1	38 kDa
	Actin muscle	KAL5971509.1	42 kDa
	Filamin-A	KAL5967555.1	129 kDa
	Filamin-A	KAL5968259.1	118 kDa
	Tubulin beta-2 chain	KAL5969386.1	50 kDa
	Tubulin alpha-1B chain	KAL5963602.1	54 kDa
	Glyceraldehyde-3-phosphate dehydrogenase	KAL5965465.1	36 kDa
Multi-pass transmembrane protein	Tetraspanin-6	KAL5962439.1	27 kDa
	Tetraspanin-7	KAL5970367.1	25 kDa

### TsEVs proteome reveals proteins with redox and immunomodulatory functions

Analysis of redox-related proteins identifies a diverse repertoire of antioxidant enzymes present in TsEVs (Table 3). Two isoforms of peroxiredoxin-1 and thioredoxin were identified, along with thioredoxin reductase 3 and thioredoxin peroxidase. Additional redox enzymes were also observed, including several isoforms of mu-class glutathione S-transferase, the ferritin heavy chain, aldo-keto reductase, 15-anhydro-D-fructose reductase, NADH-cytochrome b5 reductase 3, and a member of the short-chain dehydrogenase/reductase SDR family.

**Table 3:** Proteins related to redox metabolism in TsEVs

Protein	Accession	Molecular weight	Function
Peroxiredoxin 1	KAL5962011.1	17 kDa	Peroxides reduction, redox signaling
Peroxiredoxin 1	KAL5962010.1	5 kDa	
Thioredoxin	KAL5969986.1	12 kDa	Protein reduction, antioxidant defense
Thioredoxin reductase 3	KAL5971122.1	74 kDa	Thioredoxin reduction
Thioredoxin peroxidase	KAL5962013.1	22 kDa	Peroxides reduction
Glutathione S-transferase class-mu 26 kDa isozyme	KAL5961744.1	26 kDa	Glutathione conjugation, antioxidant
Glutathione S-transferase class-mu 28 kDa isozyme	KAL5969862.1	24 kDa	
Glutathione S-transferase class-mu 28 kDa isozyme	KAL5968510.1	24 kDa	
Ferritin heavy chain	KAL5965987.1	20 kDa	Iron storage, prevents Fenton reaction
Aldo-keto reductase family 1 member A1-A	KAL5962121.1	38 kDa	Redox enzyme, electron transfer
Aldo-keto reductase family 1 member B1	KAL5960474.1	19 kDa	
Aldo-keto reductase family 1 member D1	KAL5963842.1	14 kDa	
15-anhydro-D-fructose reductase	KAL5969679.1	13 kDa	
NADH-cytochrome b5 reductase 3	KAL5963016.1	36 kDa	
Dehydrogenase/reductase SDR family member 1	KAL5966503.1	38 kDa	

Finally, a set of proteins with immunomodulatory functions was also identified, including a broad repertoire of annexins (A6, A7, A8, A11, A13, and B12), calpain proteases (A and 5), 14-3-3 zeta proteins, paramyosin, and an Hsp70-binding protein 1 (Table 4).

**Table 4:** Proteins present in TsEv with immunomodulatory function

Protein	Accession	Molecular weight	Function
Annexin A6	KAL5970641.1	40 kDa	Immunomodulation; inhibits inflammation and regulates apoptosis
Annexin A7	KAL5960686.1	41 kDa	
Annexin A7	KAL5960688.1	36 kDa	
Annexin A8	KAL5967627.1	39 kDa	
Annexin A8	KAL5968959.1	38 kDa	
Annexin A11	KAL5969575.1	47 kDa	
Annexin A13	KAL5968958.1	36 kDa	
Annexin A13	KAL5960689.1	35 kDa	
Annexin-B12	KAL5966446.1	38 kDa	
Calpain-A	KAL5965764.1	93 kDa	Cytoskeletal remodeling; implicated in immune evasion
Calpain-5	KAL5970867.1	77 kDa	
14-3-3 protein zeta	KAL5969180.1	28 kDa	Signal transduction; modulates apoptosis and immune signaling
14-3-3 protein zeta	KAL5970244.1	29 kDa	
Paramyosin	KAL5966408.1	108 kDa	Inhibits complement activation; immune evasion
Hsp70-binding protein 1	KAL5969444.1	36 kDa	Chaperone; modulates immune response, anti-apoptotic

### TsEVs suppress ROS production in stimulated human PMN

**Figure 4:**
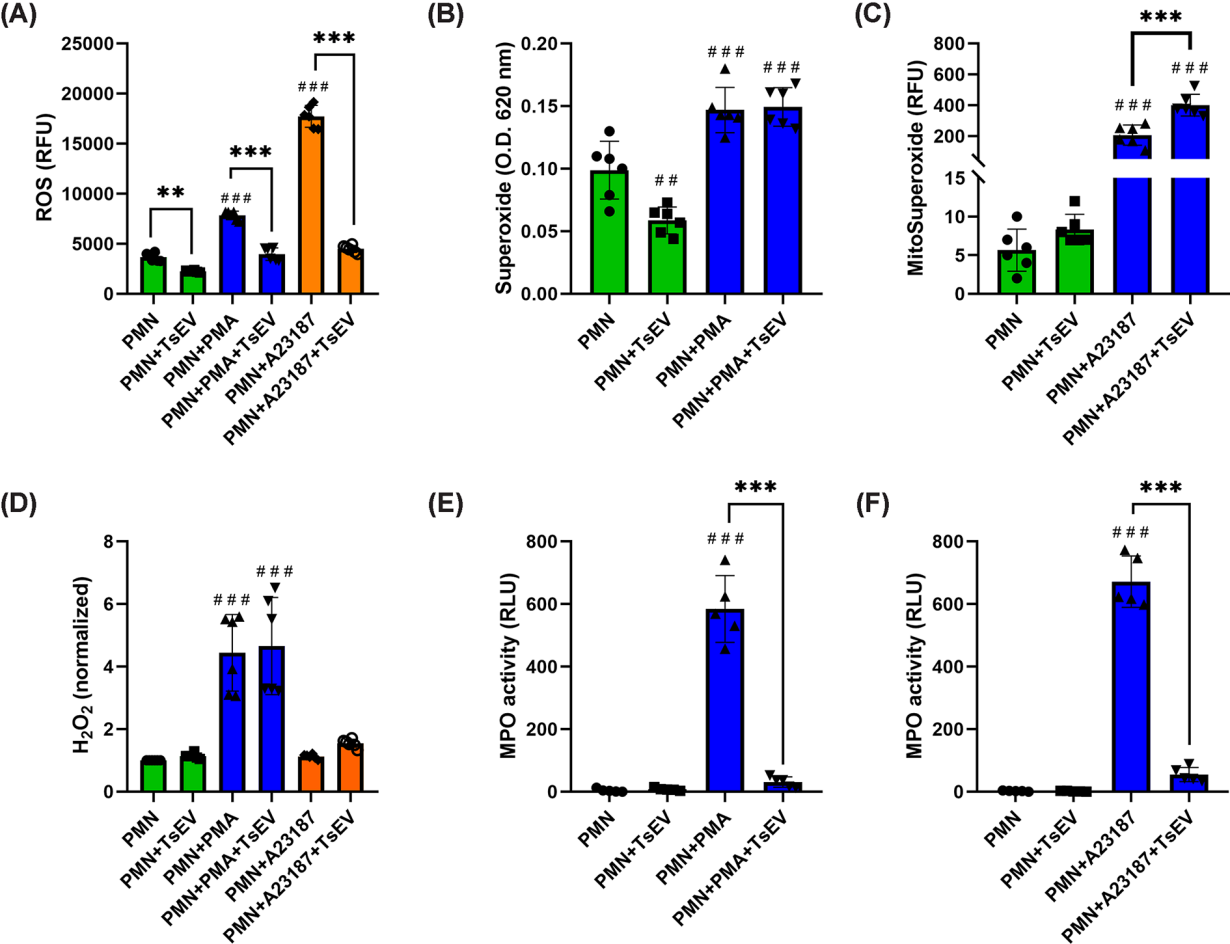
Quantification of different ROS in human PMNs treated with TsEVs (**A**) Detection of total ROS using H_2_DCFDA in human PMNs: the cells were preincubated with the fluorescent probe, placed in 96-well plates (1 × 10^5^ cells/well), treated with TsEVs (1 μg of protein), and stimulated or not with PMA (50 nM) or A23187 (10 μM). Fluorescence was measured after 1 h at 485/528 nm. (**B**) Detection of superoxide anion by NBT reduction (0.05%) assay. PMNs (1 × 10^5^ cells/tube) were treated with TsEVs (1 μg of protein) and stimulated or not with PMA (10 μM). After 2 h of incubation at 37°C, formazan crystals were solubilized, and absorbance was measured at 620 nm. (**C**) Detection of mitochondrial superoxide (MitoSuperoxide). PMNs were preincubated with MitoSOX^™^ Red, placed (1 × 10^5^ cells/well), treated with TsEVs (1 μg of protein), and stimulated or not with A23187 (10 μM). Fluorescence was measured after 2 h at 485/580 nm. (**D**) Quantification of intracellular hydrogen peroxide in PMNs. Cells were preincubated with the fluorescent sensor (30 min at 37°C), washed, placed (1 × 10^5^ cells/well), treated with TsEVs (1 μg of protein), and stimulated or not with PMA (50 nM) or A23187 (10 μM). Fluorescence was evaluated after 1 h at 485/580 nm. (**E,F**) MPO activity measured using luminol (200 μM) in PMNs (1 × 10^5^ cells/well). Cells were treated with TsEVs (1 μg of protein) and stimulated or not with PMA (50 nM) or A23187 (10 μM). Luminescence was read from the bottom of the well after 15 min (A23187) or 2.5 h (PMA). Graphs represent mean ± standard deviation. **P <0.01, ***P <0.001, and ##*P* <0.01 respect to PMN; ###*P* <0.001 respect to PMN.

It has been reported that *T. solium* cysticerci suppress the immune response at sites where they establish. Therefore, we decided to investigate the effect of TsEVs on ROS production in stimulated human PMNs. PMNs were pretreated with the general ROS indicator H_2_DCFDA and then stimulated with PMA or A23187 in the absence or presence of TsEVs. As shown in Figure 4A, TsEVs significantly reduced the basal ROS levels of unstimulated PMNs. When stimulated, PMNs produced high levels of ROS, with the calcium ionophore inducing a double level compared with PMA. Noteworthy, the addition of TsEVs to the stimulated PMNs completely suppressed ROS production to values similar to those of the unstimulated control (Figure 4A).

### TsEVs reduce basal superoxide production but not under stimulation

In order to determine the specific ROS abolished by TsEVs, the different types of ROS produced were quantified. Figure 4B shows the levels of superoxide production in PMNs stimulated with PMA in the absence or presence of TsEVs. Treatment with TsEVs significantly reduced basal superoxide production compared with unstimulated PMNs. In contrast, when stimulated with PMA, PMNs produced high levels of superoxide that were not inhibited by the addition of TsEVs, suggesting that superoxide is not the ROS affected by the nanoparticles in the stimulated cells.

### TsEVs enhance MitoSuperoxide production in PMNs treated with A23187

Figure 4C shows the levels of MitoSuperoxide in PMNs under basal conditions and following stimulation with A23187, either in the absence or presence of TsEVs. Under basal conditions, stimulation did not produce significant alterations in MitoSuperoxide levels compared with untreated PMNs. In contrast, under stimulation with A23187 that induced a marked increase in MitoSuperoxide production, the addition of TsEVs promoted that MitoSuperoxide levels increased significantly.

### TsEVs do not affect H_2_O_2_ production in previously stimulated PMNs

Figure 4D shows the normalized levels of H_2_O_2_ in PMNs under basal conditions and after stimulation with PMA or A23187, in the absence or presence of TsEVs. In this case, TsEVs treatment did not significantly affect either basal or PMA-stimulated H_2_O_2_ production in PMNs compared with untreated cells. Interestingly, stimulation with A23187 failed to produce an increase in H_2_O_2_ production in PMNs compared with unstimulated controls, and therefore, TsEVs did not modify this response either.

### TsEVs inhibit MPO activity

Figure 4E,F show the levels of MPO activity in PMNs under basal conditions and after stimulation with PMA or A23187 in the absence or presence of TsEVs. Under basal conditions, TsEV treatment did not significantly alter MPO activity compared with untreated PMNs. However, under stimulation with PMA, there resulted a significant increase in MPO activity; the addition of TsEVs markedly reduced PMA-induced MPO activity, suggesting an inhibitory effect of TsEVs on MPO activity when stimulated. Similarly, TsEVs significantly decreased MPO activity induced by A23187 when compared with untreated A23187-stimulated PMNs.

## Discussion

The study of EVs has emerged as a key area of research for understanding intercellular communication during both homeostasis and pathological conditions. In particular, parasite-derived EVs have been shown to play an important role in regulating the host’s immune system and parasite colonization and persistence, as well as the associated pathophysiology [31,32]; *T. solium* EVs obtained from porcine muscle cysticerci have previously been used to study host–parasite interactions [33]. In the present study, we describe the isolation of EVs from the racemose form of *T. solium* cysticerci (TsEVs), their proteome was analyzed, and their effect on the respiratory burst of human PMNs was evaluated. This is the first time (to our knowledge) that *T. solium* material obtained from a patient with extra-parenchymal NCC (a clinical entity that accounts for 15%–30% of NCC cases) has been used to understand the role of EVs during host–parasite interaction [28]. This biological material is extremely difficult to obtain; therefore, we are exploring the possibility of obtaining EVs using a mice model of cysticercosis based on *Taenia crassiceps* that can reproduce asexually in the peritoneal cavity of laboratory mice [34]. It remains to be seen whether these EVs result useful, considering the differences with the immunological conditions of human disease, particularly in NCC [17].

Nanotracking and TEM analyses showed that TsEVs are spherical membrane-bound particles with a modal size of 200 nm, released by both parasites for at least half a year during *in vitro* culture in RPMI medium. Longitudinal monitoring of TsEVs by nanotracking revealed a progressive increase in average size over several months in culture, ranging from 157 nm, six months after surgery, to sizes exceeding 200 nm in cultures eight months later. This variation may reflect parasite adaptation to the *in vitro* culture, affecting EV biogenesis/composition, as reported in other models in which environmental conditions alter the dynamics of EV release [35]. Proteomic analysis enabled identification of exosomal markers such as Hsp70, 14-3-3 proteins, and tetraspanins [1], which, together with GO analysis, suggested that TsEVs collected from racemose cysts under *in vitro* were primarily exosomes; however, identification of TsEVs larger than 200 nm throughout the experiment also suggested the presence of ectosomes (formerly microvesicles) [2], implying that TsEVs represent a mixed population of different EVs.

Previous studies have shown that helminth parasites release EVs as a way to evade the host immune response; for example, EVs from *T. solium* cysticerci (recovered from porcine muscle) and *T. pisiformis* have been shown to induce Th2-associated cytokine production in macrophages (an activation profile unable to eliminate the parasite) and to reduce the release of proinflammatory cytokines such as IFN-γ [33,36]. Similarly, EVs from the hydatid cyst of *E. granulosus* can block lymphocyte proliferation, decrease the production by T cells of proinflammatory cytokines IL-17 and IFN-γ, and promote the production of IL-4 [37]. Our results are consistent with this immunomodulatory profile of cestode-derived EVs, as TsEVs significantly reduced the basal production of ROS in human PMNs and completely inhibited the respiratory burst triggered by PMA (a PKC activator) and A23187 (a calcium ionophore). ROS production is one of the most important microbicidal mechanisms of PMNs, occurring primarily within the phagolysosome as an oxidative strategy to eliminate phagocytosed microorganisms [38]. However, ROS are also essential for killing pathogens that cannot be phagocytosed, where their extracellular action contributes to pathogen elimination [39]. The ability of TsEVs to block ROS formation may therefore represent a potential mechanism of immune evasion. Typically, parenchymal NCC is characterized by the absence of inflammatory infiltrate around viable cysticerci and by the appearance of a marked inflammatory reaction associated with parasite degeneration [40]. In extra-parenchymal NCC (such as the cysticerci recovered in this case), the relationship between immune response and parasite degeneration is unclear: patients with viable parasites in the cerebrospinal fluid show detectable immune responses [41], suggesting that protection is less effective. Although lymphocytes are generally considered the main immune cells involved in the response to cysticerci, some studies have reported PMNs as the predominant cell type in the cerebrospinal fluid of patients with chronic extraparenchymal NCC harboring viable parasites [24]. In this context, the capacity of TsEVs to suppress ROS production in PMNs may be relevant for escaping oxidative attack, similar to what has been documented in other parasites such as *E. granulosus*, in which *in vitro* exposure to H_2_O_2_ induces proteomic changes consistent with early apoptosis, as well as the expression of antioxidant proteins [42].

To further explore the extent to which the PMN respiratory burst was suppressed, we assessed the production of different ROS generated during this process. Interestingly, TsEVs decreased PMN basal levels of superoxide anion but were unable to reduce its levels in PMA-stimulated PMNs. Under basal conditions, superoxide production depends on a partial and transient assembly of the NADPH oxidase complex, which is highly sensitive to inhibitory signals [43]. The proteomic analysis of TsEVs did not reveal any known protein with the ability to inhibit NADPH-oxidase; however, the possibility cannot be ruled out that proteins with moonlighting activities may exist that are capable of binding to or blocking basal assembly. To date, a wide variety of moonlighting proteins have been described in cestodes (for example, *T. solium* enolases that bind plasminogen), so the presence of proteins with previously unrecognized NADPH oxidase-binding activity should not be excluded [44]. Another possibility could be the presence of low-molecular-weight molecules present in TsEVs [33], such as inositides, having the potential of regulating this process, an issue that remains to be explored [45].

Conversely, TsEVs caused an increase in mitochondrial superoxide in A23187-stimulated PMNs without affecting unstimulated PMNs. It is well established that PMNs generate mitochondrial superoxide during activation, and these mitoROS are required for effective microbicidal activity as well as for the formation of PMN extracellular traps (NETs) [46]. In this sense, TsEVs could act as a second stimulus to enhance mitochondrial superoxide production and increase the microbicidal ability of PMNs. However, this stimulus alone appears to be insufficient to initiate such a response, as resting PMNs did not exhibit an increase in mitochondrial superoxide production. It is noteworthy that TsEVs did not affect hydrogen peroxide production but completely suppressed MPO activity, whose enzymatic product, hypochlorous acid (HClO), is the most potent microbicidal ROS [47]. Notably, ROS quantification using H_2_DCFDA reflects the global intracellular oxidative environment. This molecule can be oxidized by different oxidants, including those generated during MPO catalytic cycle. In particular, the highly oxidizing intermediates MPO Compound I and Compound II are capable of promoting its oxidation, and HClO, the principal MPO-derived product, may also contribute to the fluorescent signal [48]. Therefore, a reduction in MPO activity could indirectly reflect a lower H_2_DCFDA signal, as we observed in this work. Nevertheless, more selective probes and enzymatic inhibition strategies will be required to define the precise contribution of MPO to the ROS decrease. Importantly, TsEVs were able to inhibit the formation of HClO, which has been shown to kill cestode parasites such as *E. granulosus* [42]. It is worth mentioning that we observed this ROS decrease using 1 μg of EV-associated protein for the in vitro assays. This concentration is tenfold lower than that previously reported by our group, in which a reduction in ROS production was observed in chemically stimulated PMNs treated with *E. histolytica* EVs [10]. Importantly, the use of low-microgram EV doses is also consistent with *in vivo* studies of pathogen-derived EVs, where biological effects have been reported following administration of ∼2 μg of protein-associated EVs administered consecutively over time [49]. These works suggest that the effects observed here are not the result of supraphysiological exposure.

Proteomic analysis of TsEVs revealed the presence of several redox-related proteins, including peroxiredoxin, thioredoxin, thioredoxin reductase, and thioredoxin peroxidase. These enzymes can detoxify ROS, contributing to cellular redox balance but also functioning as immune evasion mechanisms in parasites by neutralizing exogenous ROS generated by immune cells [50]. Although these enzymes detoxify peroxides, we did not observe changes in hydrogen peroxide levels; thus, their presence in TsEVs does not necessarily indicate functional activity within the recipient cell (PMNs in this case), and mechanistic assumptions based solely on EV proteomics must be experimentally validated. Interestingly, the TsEVs proteome also revealed the heavy chain of ferritin, a protein capable of sequestering Fe^2+^ [51], which participates in the Fenton reaction together with H_2_O_2_ to generate hydroxyl radicals, highly reactive species that oxidize proteins as well as DNA and constitute a secondary product of the respiratory burst [47,52]. H_2_DCFDA probe used in the present study also reacts with this ROS type, which may partially explain the observed reduction in ROS levels [53]. Ongoing work in our group aims to determine the contribution of this protein to ROS production in PMNs.

A limitation of the present study is the number of racemose cysticerci analyzed, as TsEVs were obtained from two parasites derived from the same patient. Therefore, the data reported in this work should be interpreted as representative of these specific specimens. Further studies including parasites from independent patients will be necessary to determine inter-individual variability; identify the specific molecules responsible for the observed effects; determine whether TsEVs exert similar effects on other innate immune cells, such as macrophages, or on adaptive immune cells, such as T lymphocytes; and assess additional immunological consequences mediated by TsEVs. Likewise, *in vivo* evaluation of the functional impact of these vesicles will allow assessment of their pathophysiological relevance beyond the cellular context.

## Data Availability

The mass spectrometry proteomic dataset supporting the present study has been deposited in Zenodo repository under the DOI: https://doi.org/10.5281/zenodo.17887554 [54].

## Abbreviations

ACN: acetonitrile
EVs: extracellular vesicles
MPO: myeloperoxidase
NBT: Nitroblue tetrazolium
NCC: neurocysticercosis
NTA: nanoparticle tracking analysis
PBS: phosphate buffer solution
ROS: reactive oxygen species
TEM: transmission electron microscopy
TFA: trifluoroacetic acid
TsEVs: *Taenia solium* extracellular vesicles
